# HPV infection related immune infiltration gene associated therapeutic strategy and clinical outcome in HNSCC

**DOI:** 10.1186/s12885-020-07298-y

**Published:** 2020-08-24

**Authors:** Hao Zeng, Xindi Song, Jianrui Ji, Linyan Chen, Qimeng Liao, Xuelei Ma

**Affiliations:** 1 Department of Biotherapy, Cancer Center, West China Hospital, Sichuan University, No. 37, GuoXue Alley, Chengdu, 610041 People’s Republic of China; 2 State Key Laboratory of Biotherapy and Cancer Center, West China Hospital, Sichuan University and Collaborative Innovation Center for Biotherapy, Chengdu, China; 3 West China School of Medicine, West China Hospital, Sichuan University, Chengdu, China

**Keywords:** HNSCC, Tumor microenvironment, Gene, HPV, Immunotherapy

## Abstract

**Background:**

Head and neck squamous cell carcinoma (HNSCC) is the sixth most common tumor in human. Research has shown that HPV status HNSCC is a unique prognosis factor, which may due to its immune infiltration landscape. But the underlying mechanism is unclear.

**Methods:**

In this study, we used a combination of several bioinformatics tools, including WCGNA, ssGSEA, CIBERSORT, TIDE,etc., to explore significant genes both related to HPV infection status and immune cell infiltration in HNSCC patients.

**Results:**

Combined with several bioinformatics algorithms, eight hub genes were identified, including LTB, CD19, CD3D, SKAP1, KLRB1, CCL19, TBC1D10C and ARHGAP4. In HNSCC population, the hub genes had a stable co-expression, which was related to immune cell infiltration, especially CD8+ T cells, and the infiltrative immune cells were in a dysfunctional status. Samples with high hub genes expression presented with better response to immune check point block (ICB) therapy and sensitivity to bleomycin and methotrexate.

**Conclusions:**

The eight hub genes we found presented with a stable co-expression in immune cell infiltration of HPV + ve HNSCC population. The co-expression of hub genes related to an immune microenvironment featuring an increase in immune cells but high degree of immune dysfunction status. Patients with high hub gene expression had a better response to ICB treatment, bleomycin and methotrexate. The co-expression of hub genes may be related to immune infiltration status in patients. The concrete molecular mechanism of hub genes function demands further exploration.

## Background

Head and neck squamous cell carcinoma (HNSCC) is the sixth most common tumor in human [1], numbering eighth in the list of causes of tumor-related death [2] and accounting for over 500,000 new cases each year worldwide [3, 4].

The two traditional main risk factors for HNSCC are alcohol and smoking, while the past decades witnessed an increasing population of HNSCC patients with persistent infection of human papillomavirus (HPV) [5, 6], which is lately responsible for 60–80% of the oropharyngeal cancer incidence in the United States and Europe [7–9]. Patients who develop HPV + ve HNSCC generally end up with a better prognosis when compared to patients with HPV-ve HNSCC [10–13]

Previous studies have demonstrated that immune system plays a significant role in the development of HNSCC [5], and there are significant differences in the composition of tumor microenvironment (TME) [14–16] between HPV-infected and non-HPV-infected HNSCC. TME, consisting of tumor tissue, micro vessels, cytokine and tumor-infiltrating immune cells (TICs) etc., are essential for tumor progression. Differences in the compositions of TICs, including cytotoxic T cells, helper T cells, dendritic cells (DCs), as well as related inflammatory pathways lead to different clinical tumor behavior [17]. However, immune landscape and its interaction with HPV status is still unclear.

Currently, the majority of patients are commonly provided with the standard treatment of surgery, radiotherapy, chemotherapy, or a combination of these therapies, but recurrence and resistance to following treatment will occur in about 40–60% of treated patients [16]. Therefore, the 5-year OS rate of HNSCCs hasn’t changed much over the past decade [16]. Studies on the immune infiltration feature and underlying molecular mechanism of HNSCC population may provide potential molecular targets that improve therapeutic selection and better predict the therapeutic response.

With the development of bioinformatics tools, it is possible to process a huge scale of data at one time. In terms of immune system, a great quantity of algorithms has emerged, such as Estimation of STromal and Immune cells in Malignant Tumor tissue Expression (ESTIMATE), Single sample Gene Set Enrichment analysis (ssGSEA), Cibersort, Tumor Immune Dysfunction and Exclusion (TIDE),etc. Weighted gene co-expression network analysis (WGCNA) has been applied in various types of tumor, in which it is used to look for interaction between gene expression features and clinical characteristics. ESTIMATE is used to assess the extent of immune cells and stromal cells infiltration of tumor tissue by gene expression signatures.

In this study, we obtained HNSCC samples containing both HPV status and sequencing data from public available databases. Combined with the algorithms mentioned, we aimed to identify hub genes associates with immune cell infiltration and HPV status in HNSCC population. We also assessed the infiltrative immune cell type and function, willing to explore the effect of hub genes on immune microenvironment of this specific tumor subtype. In addition, we analyzed the response to immune check point treatment and drug sensitivity in samples with high hub genes expression.

## Methods

### Data procession of gene expression omnibus

We downloaded two gene expression microarrays (GSE65858 and GSE40774) of HNSCC from NCBI Gene Expression Omnibus (GEO; https://www.ncbi.nlm.nih.gov/geo/). GSE65858 contained 268 samples (73 HPV + ve, 196 HPV-ve), and GSE40774 contained 134 (58HPV + ve, 76 HPV-ve) samples. We used R package limma to normalize the data and screen the differently expressed genes (DEGs).

### Data procession of TCGA

We filtered the clinical characteristics and gene expression data of the Cancer Genome Atlas Project database (TCGA, https://cancergenome.nih.gov/) HNSCC cohort, in which we collected 102 samples with both HPV status imformation and sequencing data (30 HPV + ve, 72 HPV-ve). We used Variance Stabilizing Transformation function of R package DEseq2 to normalize data and do differential expression analysis between HPV-infection and none-HPV-infection group.

### Immune microenvironment assessment

Among TICs, the two major type of cells are immune and stromal cells. Immune and stromal scores were calculated by analyzing specific gene expression signature, and combined to represent a measurement of tumor purity. In this study, we used R package ESTIMATE [18] to assess the immune infiltration of HNSCC samples.

We implemented CIBERSORT [19] (http://cibersort.stanford.edu/) to quantify the tumor infiltrating lymphocytes (TILs) in HNSCC samples. CIBERSORT is a deconvolution algorithm. By using signature matrix (referring to gene expression values), which minimally represents each cell type, as well as support vector regression, it measures the population of each type of cell from bulk tumor samples. We uploaded the standardized gene expression matrix to the website of CIBERSORT (mentioned above), then ran the algorithm under LM22 signature and 1000 permutations. The LM22 gene signature includes over 500 genes which features high sensitivity and specification of common human immune cell types such like neutrophils, NK cells, DCs, macrophages, T cells, eosinophils, B cells, etc. Patients with *P* value under 0.05 were enrolled for further analysis.

We then quantified the infiltration levels of immune cell types by ssGSEA in R package gsva [11, 20]. The ssGSEA uses the scoring result to individual tumor samples. In our study, we enrolled 24 immune cells of both innate immunity and adaptive immunity. We further analysised the function status of TILs by Tumor Immune Dysfunction and Exclusion (TIDE) algorithm [21].

### Weighted co-expression network construction

The gene expression microarray GSE65858 was further used for WGCNA. We selected the top 25% genes (ranked by SD from high to low) to construct co-expression network. We calculated the adjacency coefficient (aij) through R package WGCNA, which represented the association extent between each two genes as follows:

$$ {\mathrm{S}}_{\mathrm{ij}}=\left|\mathrm{cor}\left(\mathrm{xi},\mathrm{xj}\right)\right|{\mathrm{a}}_{\mathrm{ij}}={{\mathrm{S}}_{\mathrm{ij}}}^{\upbeta} $$

Supposing that X_i_ and X_j_ refer to expression value vectors for gene i and j, s_ij_ refer to the Pearson’s correlation coefficient of X_i_ and X_j_, and is exponentially transformed into a_ij_, which measured the network association extent between gene i and j. The soft-thresholding borderline was set by soft threshold function of R package WGCNA to construct a scale-free network. The power of β = 4 (scale-free *R*^*2*^ = 0.95) was set as the parameter of soft-thresholding. We gathered genes with high absolute value of correlations in one module in the network. We calculated the topological overlap measure (TOM) by the following equation:

$$ {\mathrm{TOM}}_{\mathrm{i},\mathrm{j}}=\frac{\sum_{K=1}^N Ai,k\ast Ak,j+ Ai,j}{\min \left( Ki, Kj\right)+1- Ai,j} $$

Modules are identified through hierarchical clustering of the weighting coefficient matrix. TOM refer to the overlap in neighboring genes of i and j.

### HPV/immune-associated key modules and gene signature identification

We analyzed the relation between modules and their clinical or genetic features in order to identify the HPV/immune-associated key modules. We first assessed the relation between module eigengenes (MEs) and certain feature. ME is the first principal component of each module, of which the expression represents all genes in the module. Then, we calculated the gene significance (GS) respectively for each gene (GS = lgP) in the linear regression between sample features and gene expression. Then we calculated the module significance (MS), referring to the average GS of all genes in a module. We selected the module with highest absolute MS value to be the most relevant module of selected sample features.

We downloaded the single cell sequencing data of profile GSE103322 and gene signatures of tumor-related pathways from CancerSEA (http://biocc.hrbmu.edu.cn/CancerSEA/). GSE103322 included 2105 tumor cells. The expression of tumor-associated pathways (including apoptosis, angiogenesis, differentiation, cell cycle, DNA damage, EMT (epithelial to mesenchymal), DNA repair, inflammation, invasion, quiescence and stemness pathways) in tumor cell was analyzed by through Gene Set Enrichment Analysis (GSEA). We applied k-NN (k = 15) algorithm from R package “FNN” to calculate the nearest neighbor of each cell at gene expression level and generate the nearest neighbor graph. The neighbors of each cell were taken as input for the visualization. Fruchterman Reingold algorithm was applied to calculate the force-directed layout on the k-NN graph via Gephi Toolkit. The red color referred to the pathway with high expression in the cell, while blue color referred to a low expression.

Hub genes correlated with HPV-infection and immune infiltration microenvironment were expected to be candidate genes. We identified the hub genes by two steps. Firstly, in GSE65858, the module membership (MM) of each gene in key module was calculated. The genes with MM over 0.8 was considered to be associated with the module features, as is described above. Secondly, we used R package limma for GSE40774 and DEseq2 for TCGA HNSCC to do differential expression analysis respectively. The cut off value was set as log2FC > |1|, and adjusted *P*-value < 0.01. We picked the intersection between the results from GSE65858, GSE40774 and TGCA database, among which we identified top eight hub genes. Differential expression analysis of hub genes was performed between tumor and normal samples, and also among different grade of tumor.

### Prediction of therapy response

The expression degree of hub genes in GSE65858, GSE40774 and TCGA HNSCC were selected for further analysis. We used GEPIA2 (http://gepia2.cancer-pku.cn/) to anlyze survival outcome of hub genes; P under 0.05 was considered to be statistically significant.

Immune checkpoint blockade (ICB) is a novel tumor therapy, but the effective population is still unsure. To measure ICB response, we used the TIDE algorithm and subclass mapping. Two immune blockade drugs, CTLA4 and PD-1, were chosen. Besides, in order to explore whether hub genes could add information to exsisting criteria in therapy chosen, we applied a multivariate analysis. We firstly divided the patients into 6 subgroups(p16 positive/negative, CD274 score high/low, immune cell score high/low). Then we divided each subgroup into two groups based on the expression of these hub genes. Finally, we applied subclass mapping algorithm and pRRophetic algorithm for each subgroup in three independent datasets to test the independency and robustness of the hub gene marker.

We predicted the chemotherapeutic response of our samples on the pharmacogenomics database [the Genomics of Drug Sensitivity in Cancer (GDSC), https://www.cancerrxgene.org/]. Four widely used chemotherapeutic agents for HNSCC, bleomycin, docetaxel, methotrexate, cisplatin, were selected. We used R package “pRRophetic” for prediction. The half-maximal inhibitory concentration (IC50) of the samples, as the parameter, was calculated by ridge regression. Rediction accuracy was assessed by 10-fold cross-validation, basing on the GDSC training set. Subgroup analysis was performed to test the independency and robustness of the hub gene marker in chemotherapy. The subgroups chosen was immune cell score high/low, HPV + ve/−ve and CD274 score high/low.

## Results

### Weighted co-expression network construction and key modules identification

**Fig. 1:**
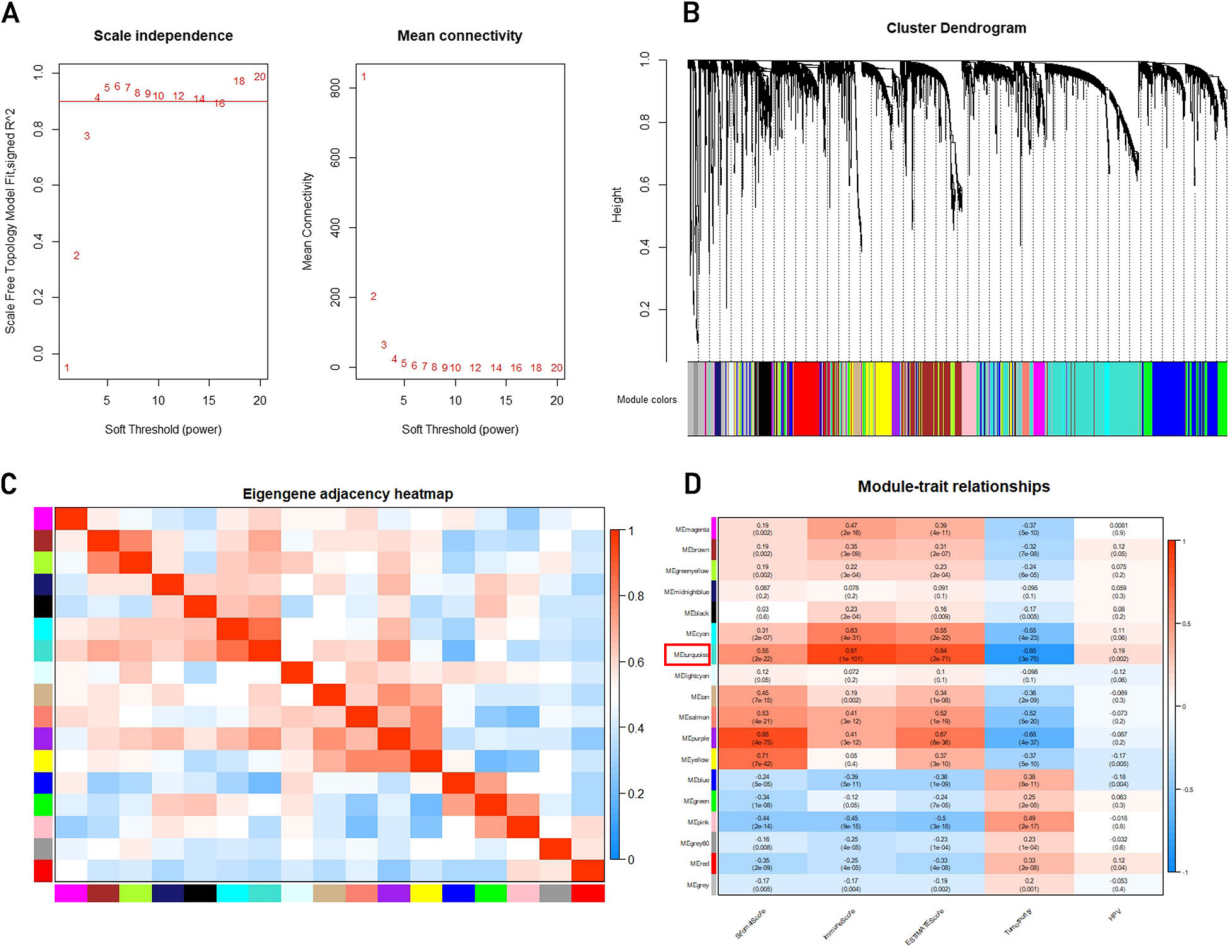
Construction of co-expression modules and identification of clinically significant modules. **a**) Analysis of the scale-free fit index and mean connectivity for different soft thresholding powers, and four was the most fit power value. **b**) The cluster dendrogram of top 25% genes ranked by SD from large to small in GSE65858. Each branch represents one gene, and each color represents one co-expression module. **c**) Heatmap of the correlation between module eigengenes (MEs) and the clinical features of HNSCC samples. **d**) The turquoise module was selected as the most significant module for further analysis

After processing the raw data, we performed WCGNA in order to screen out the genes related with both HPV infection status and immune cell infiltration. Initially, we clustered the samples GSE65858 by average linkage method, and no outsider sample was found (Fig. 1b).

Then we selected the soft-thresholding power to develop a scale-free network. Figure 1a showed that through thresholding powers from 1 to 20, we measured the network topology, and selected the mean connectivity and balanced scale. Then, the power of β =4 (scale-free *R2* = 0.95) was selected. We finally identified 17 modules in total (Fig. 1c) and we removed genes in grey which did not belong to any modules. Therefore, we selected the turquoise module which had the most significant relation with Immune score and HPV infection status for further analysis (Fig. 1d).

**Fig. 2:**
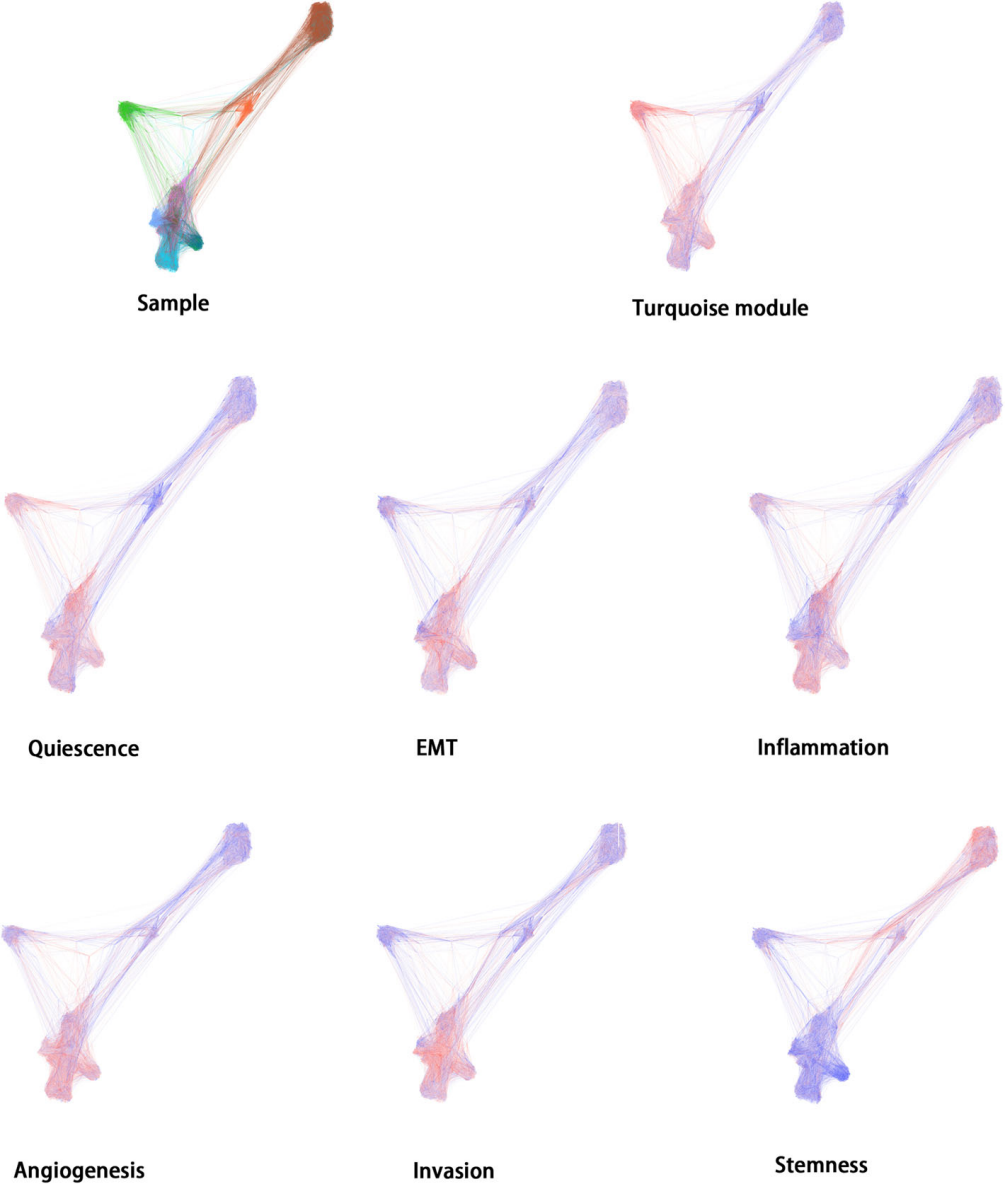
We downloaded the single cell sequencing data of profile GSE103322 and gene signatures of several tumor-related pathways from CancerSEA. The expression of tumor-associated pathways (including apoptosis, angiogenesis, differentiation, cell cycle, DNA damage, EMT, DNA repair, inflammation, invasion, quiescence and stemness pathways) in tumor cell was analyzed by through Gene Set Enrichment Analysis(GSEA). The red color referred to the pathway with high expression in the cell, while blue color referred to a low expression. The expression of turquoise module was related to some critical cell signaling pathways

In order to further validate the function of genes in turquoise module, we downloded single cell sequencing data profile GSE103322, including 2105 HNSCC tumor cells, and evaluated the performance of tumor-related pathways (including apoptosis, angiogenesis, differentiation, cell cycle, DNA damage, EMT, DNA repair, inflammation, invasion, quiescence and stemness pathways) through Gene Set Variation Analysis (GSVA). The overall view of all samples was shown in Fig. 2, including the expression of turquoise module and the expression of different tumor-related pathways respectively. It was shown that in samples with high turquoise module expression, the quiescence pathway expressed highly as well. The angiogenesis, differentiation, EMT, inflammation and invasion pathway were also significantly expressed in samples with high turquoise module expression.

### Hub genes identification

To further identify the candidate genes in turquoise module, we defined MM over 0.8 and GS of immune infiltration over 0.5 as the cutoff threshold. We then identified 127 candidate genes in total.

**Fig. 3:**
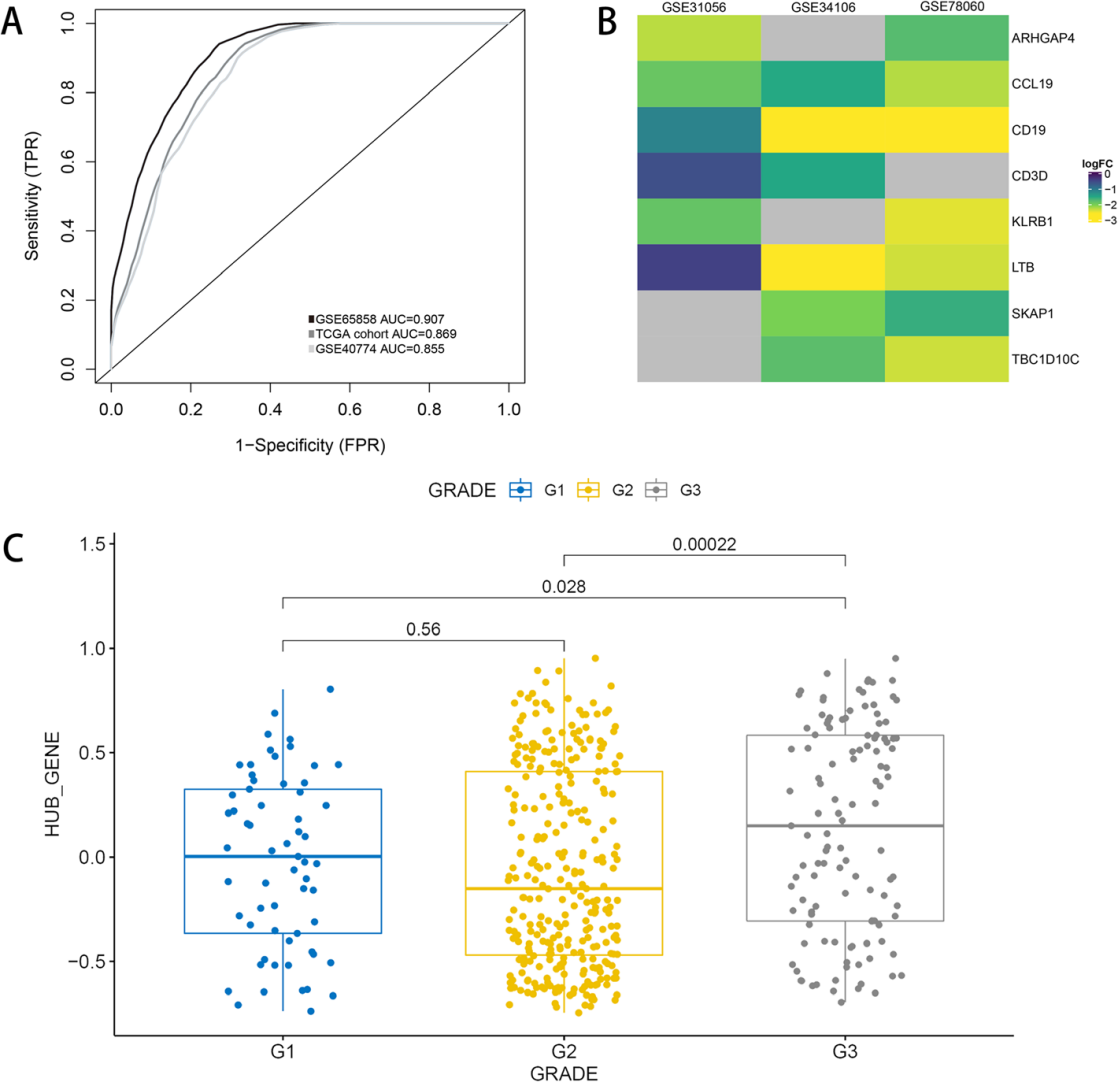
**a**) ROC showed that high expression of hub genes were closely related to HPV infection status. **b**) Hub genes were highly expressed in normal samplescompared with HNSCC samples (grey represents no statistical significance). **c**) Hub genes were highly expressed in high grade HNSCC

To screen out the genes significantly related to HPV infection, we did differential expression analysis in two independent cohorts (TCGA HNSCC, GSE40774) between HPV + ve and HPV-ve samples (log2FC > |1|and adjusted *P*-value < 0.01). Then, we selected the intersection of genes in turquoise module and DEGs. In total we got 8 genes that was significantly associated with immune infiltration in HPV + ve HNSCC population, including LTB, CD19, CD3D, SKAP1, KLRB1, CCL19, TBC1D10C, ARHGAP4. The receiver operating characteristic curve showed that these hub genes were closely related to HPV status. (Figure 3a) hub genes were found to be highly expressed in normal samples compared with tumor samples (Fig. 3b). Besides, these gene signature were significantly highly expressed in high grade HNSCC (Fig. 3c).

**Fig. 4:**
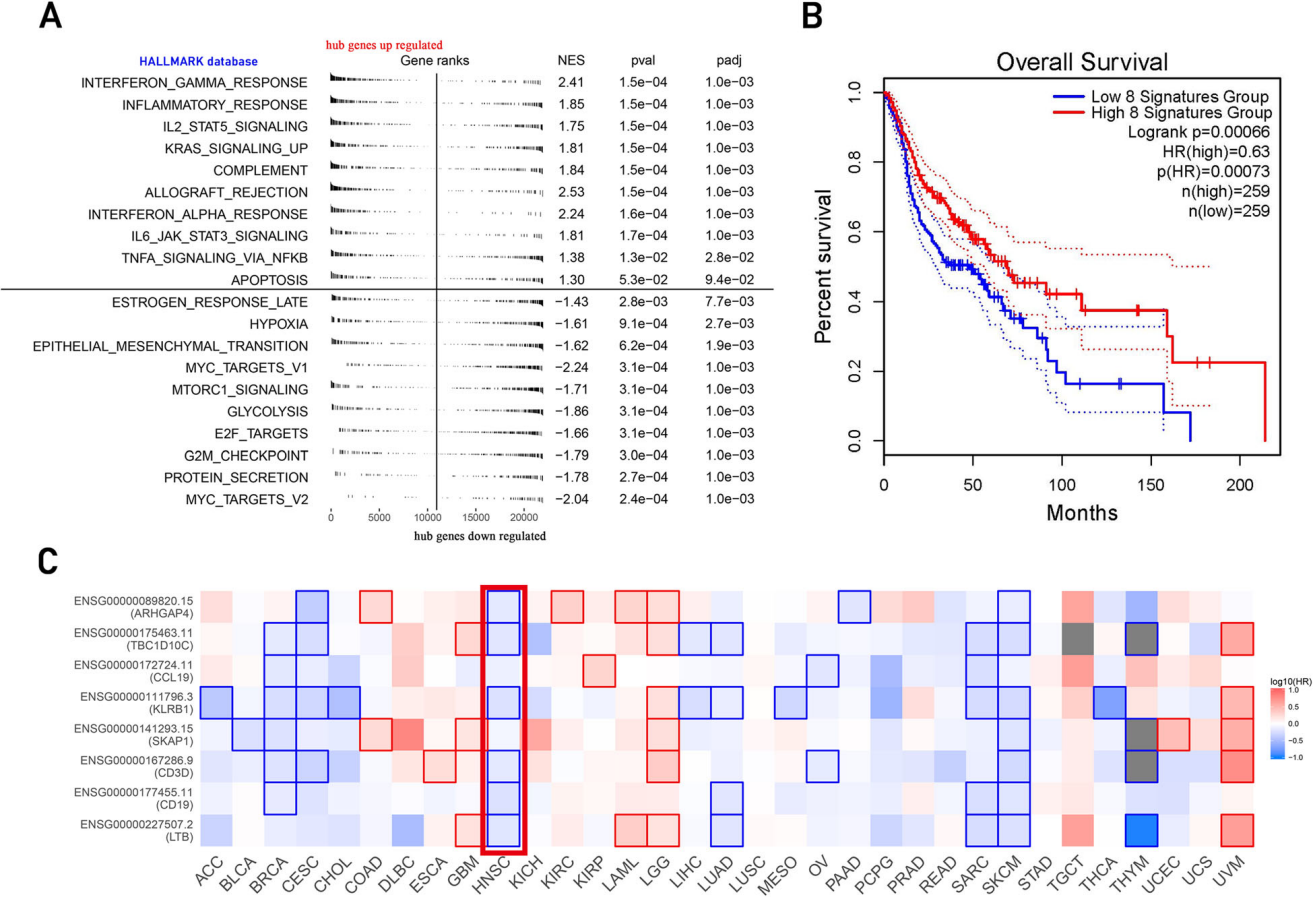
**a**) Gene Set Enrichment Analysis(GSEA) based on HALLMARK database. The result showed that the expression of several immune-related pathways were significantly upregulated in the group with high expression level of hub genes. **b**) The overall survival of high and low hub genes expression **c**) The expression of hub genes may be related to the better prognosis of HNSCC. The outlined square indicated a significant P value. The red rectangle showed the result of HNSCC population

**Fig. 5:**
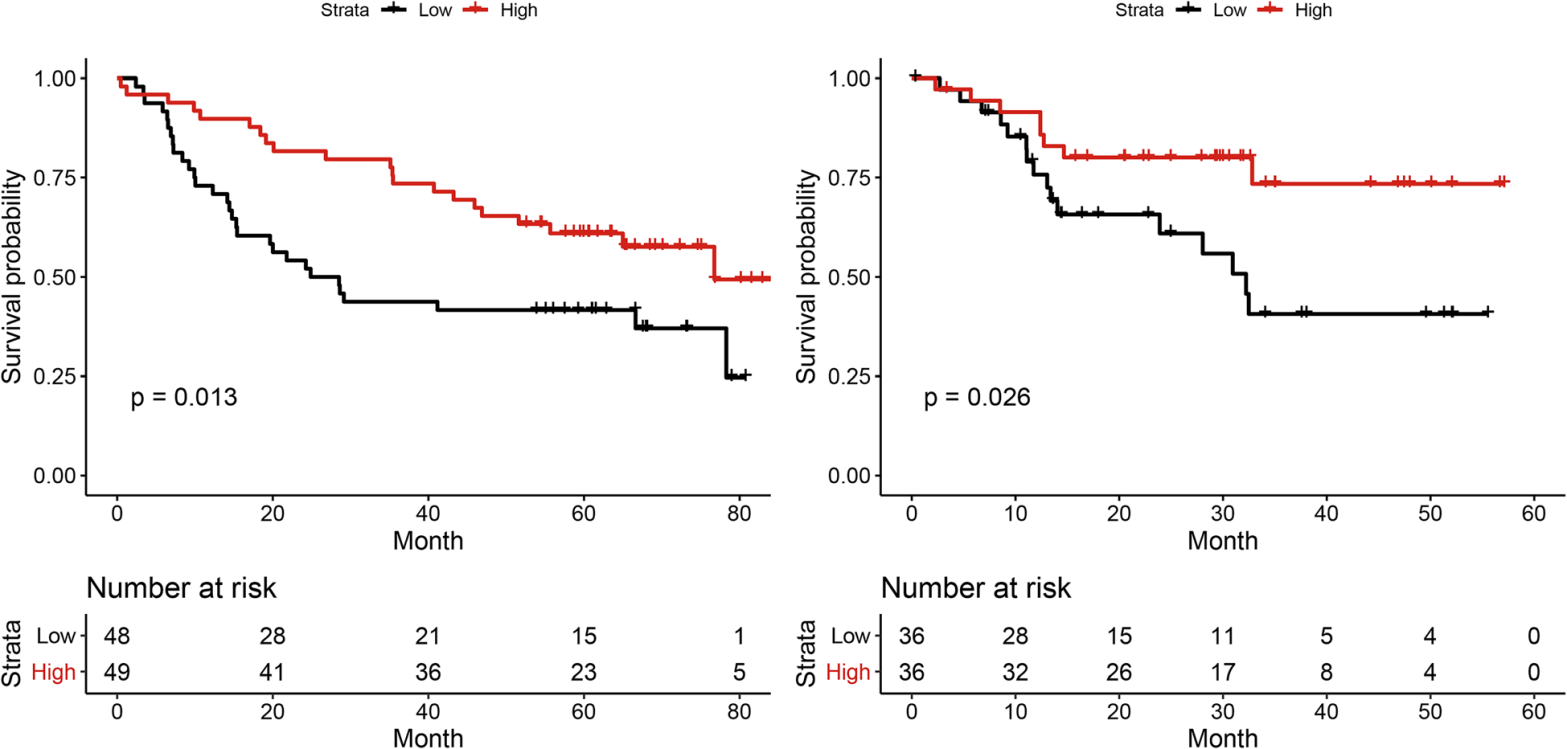
In the HPV negative cohort, eight hub genes had significant correlation with prognosis in two independent cohorts (*p* <0.05). Hub gene could be independently related to a better prognosis

The overall survival of high and low hub genes expression is shown in Fig. 4b. Since the expression of these genes could be different with a certain HPV status, we further explored independancy of the hub gene in prognosis. In the HPV negative cohort, these hub genes are significant correlation with prognosis in two independent cohorts (*p* < 0.05) (Fig. 5). To sum up, the high expression of hub genes could be related to the better prognosis (Fig. 4c).

### Hub genes were highly associated with tumor immune microenvironment

**Fig. 6:**
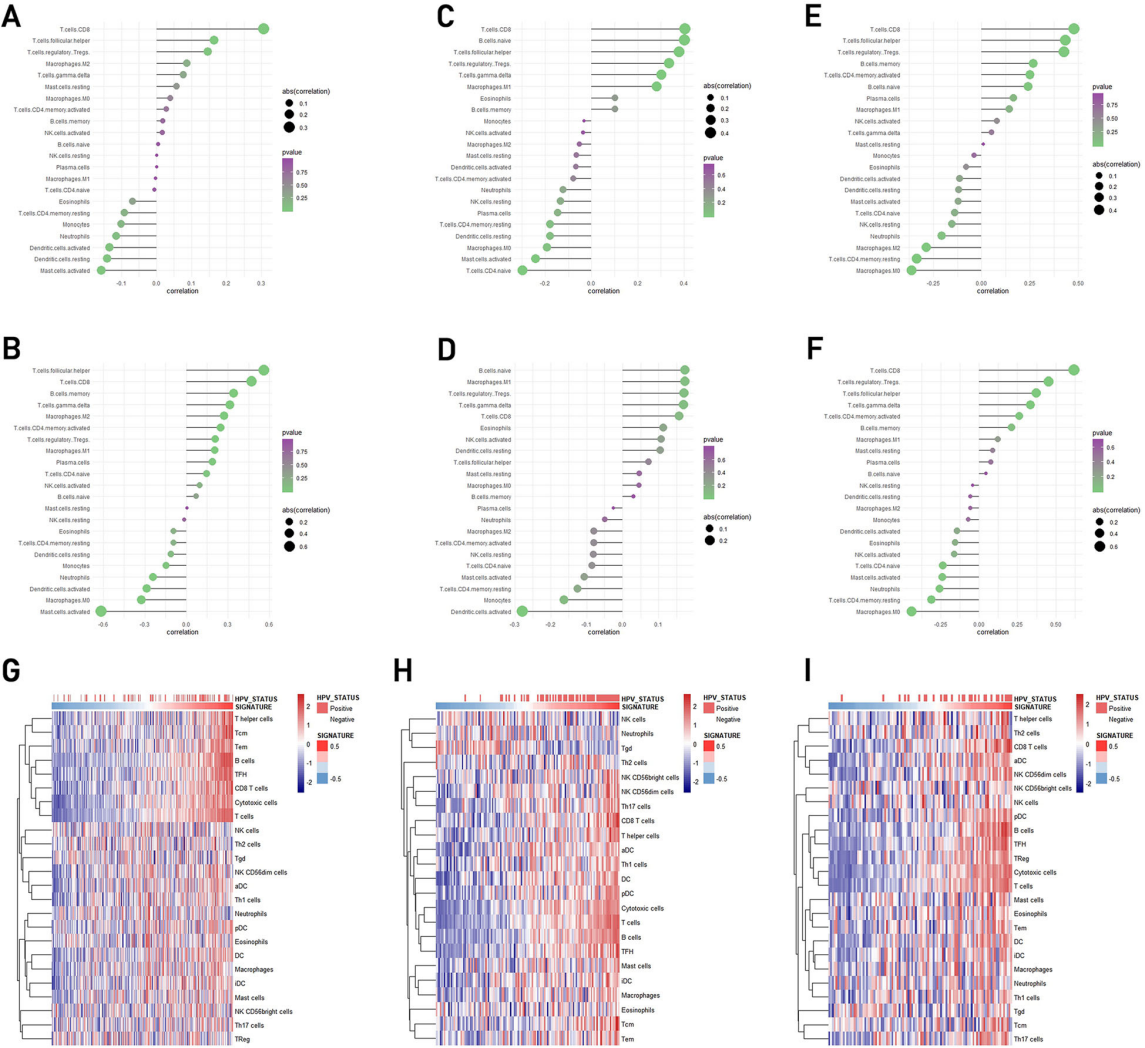
**a**-**f**) The infiltrative immune cell type of HNSCC samples with HPV infected (upper row) and high hub gene expression (down row) shared a similar result, especially CD8+ T cell. **a**) In GSE65858, correlation between TILs and HPV status. **b**) In GSE65858, correlation between TILs and hub gene expression. **c**) In GSE40774, correlation between TILs and HPV status. **d**) In GSE40774, correlation between TILs and hub gene expression. **e**) In TCGA HNSCC, correlation between TILs and HPV status. **f**) In TCGA HNSCC, correlation between TILs and hub gene expression. **g**-**i**) Heatmap of ssGSEA algorithm showed that hub genes are in positive correlation with both HPV infection status and hub genes in three data sets

In order to further explore the infiltration landscape immune cells in HNSCC population with hub genes expression and HPV infection, we used the Gene Set Variation Analysis (GSVA) and CIBERSORT algorithm. To comprehensively assess the expression of the eight hub genes, we used GSVA to create a gene signature of them, and then used CIBERSORT to measure the infiltration of TILs. The result was shown in Fig. 6. In HNSCC population with high hub genes expression, the mostly infiltrative immune cells were CD8+ T cell, Treg, macrophage M1 and naïve B cell (Fig. 6a-f). And the most common immune cell types in HPV infected HNSCC population are CD8+ T cell, follicular helper T cell, Treg and γδT cell. In HPV-ve samples, the following types of immune cell are highly infiltrated: activated mast cell, resting dendritic cell, activated dendritic cell, resting CD4+ T memory cell (Fig. 6a-f). There was a great similarity of immune cell infiltration type in HPV + ve and high hub genes expression cohorts in form of cell infiltration, especially CD8+ T cell, indicating that hub genes expressionwere closely related to immune cell infiltration of HNSCC population.

To further validate the result, we used ssGSEA to calculate the relation among hub gene expression, infection status and infiltration of different immune cells in HNSCC patients. The ssGSEA outcome showed a high positive association between the three aspects (Fig. 6g, h, i), and the most relevant cells were T cells.

To sum up, hub genes expression may indicate a positive relation to immune cell infiltration, such as CD8+ T cell, follicular helper T cell, Treg and γδT cell, especially CD8+ T cell. The outcomes are similar and stable among different sample sets and methods.

**Fig. 7:**
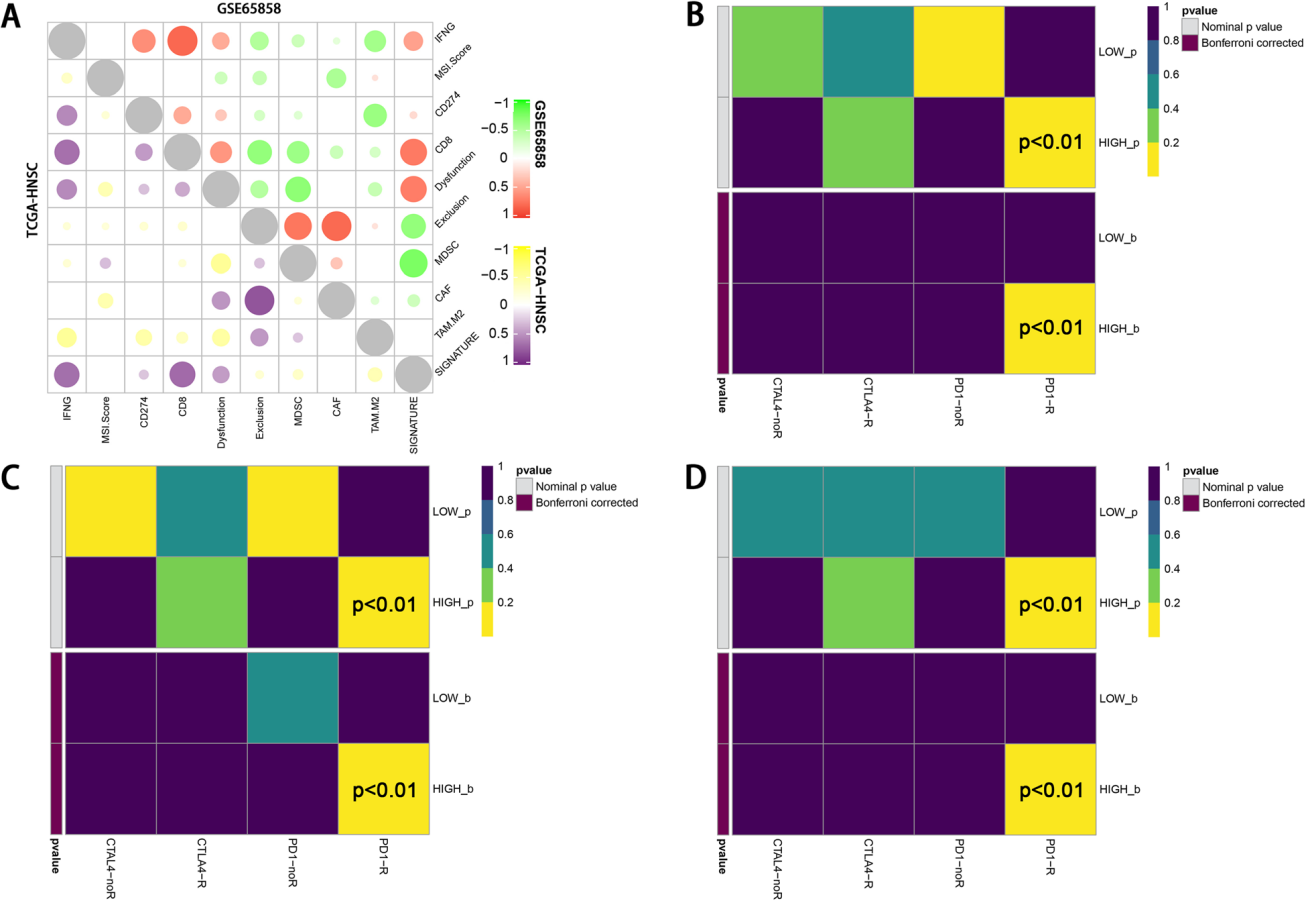
**a**) Correlation analysis by TIDE algorithm showed a positive association with T cell dysfunction in tumor and hub gene expression. The TIDE result also showed that the expression of hub genes was significantly associated with the activation of CD274 (PD-L1) **b**, **c**, **d**) PD1-R referred to patients with good response to PD1/PD-L1 therapy, while PD1-noR referred to patients with limited response to this therapy. The longitudinal axis showed patients with high and low hub gene expression. In each figure, the two lines at the bottom were the adjusted results of the upper two lines. The subclass mapping algorithm showed that patients with high hub gene expression had a similarity in genome with patients in PD1-R groups (*p* <0.01), indicating that patients with high hub gene expression may had better response to PD-1/PD-L1 therapy. This relation was consistent across all three data sets

We then performed TIDE algorithm to further explore the function of the TILs and the result was shown in Fig. 7a. We found that the expression of hub genes had a positive association with immune dysfunction in tumor (*P* < 0.01, correlation coefficient was 0.46).

### Therapeutic strategies innovation

**Fig. 8:**
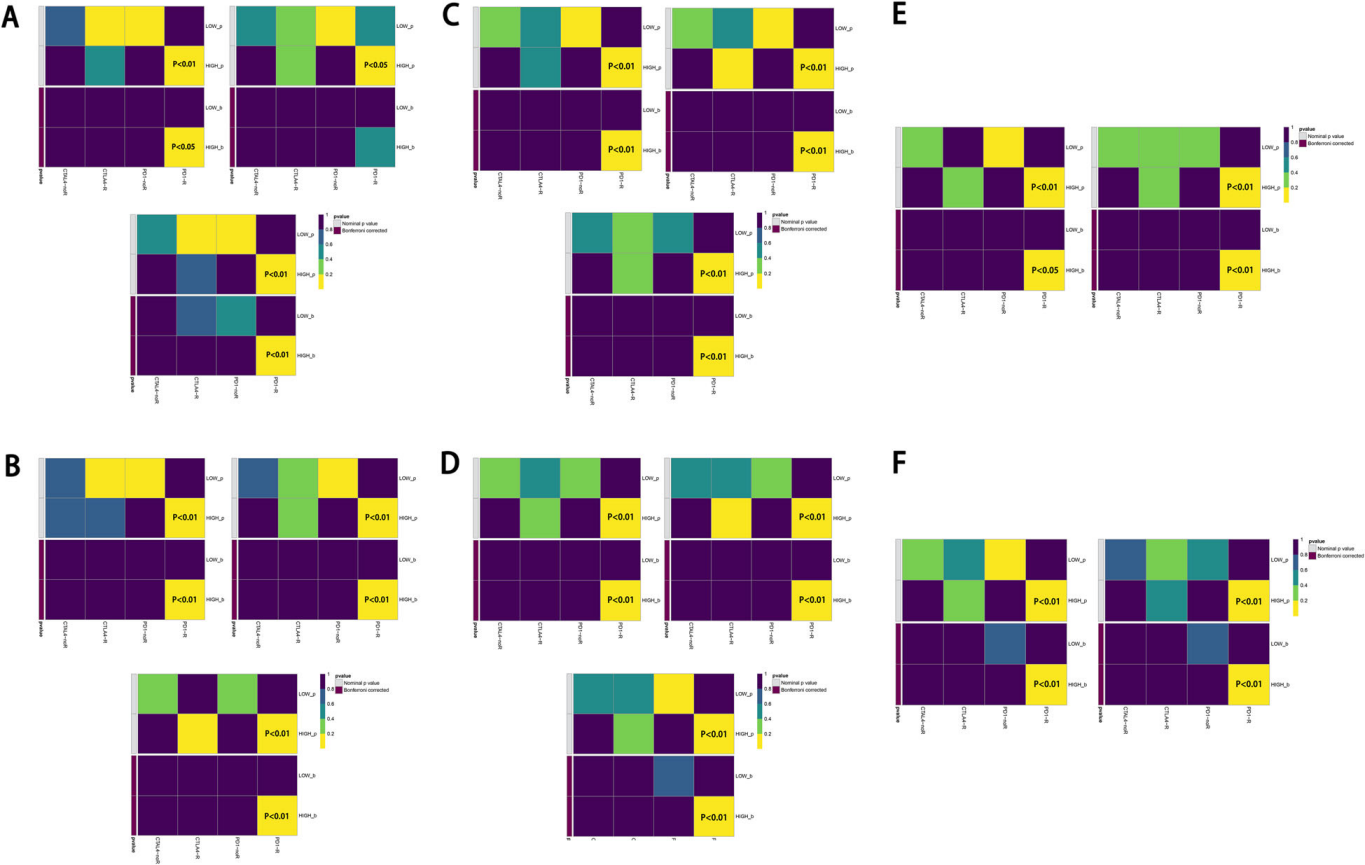
The result of multivariate analysis showed that these hub gene marker have a stable performance in ICB prediction regardless of exsisting criteria. This result was consistent across all three data sets. **a**) Subgroup of PDL-1 combined tumor and immune cell score (+). **b**) Subgroup of PDL-1 combined tumor and immune cell score (-). **c**) Subgroup of p16 positivity (+). **d**) Subgroup of p16 positivity (-). **e**) Subgroup of PDL-1 Tumor Proportion Score (+). **f**) Subgroup of PDL-1 Tumor Proportion Score (-)

**Fig. 9:**
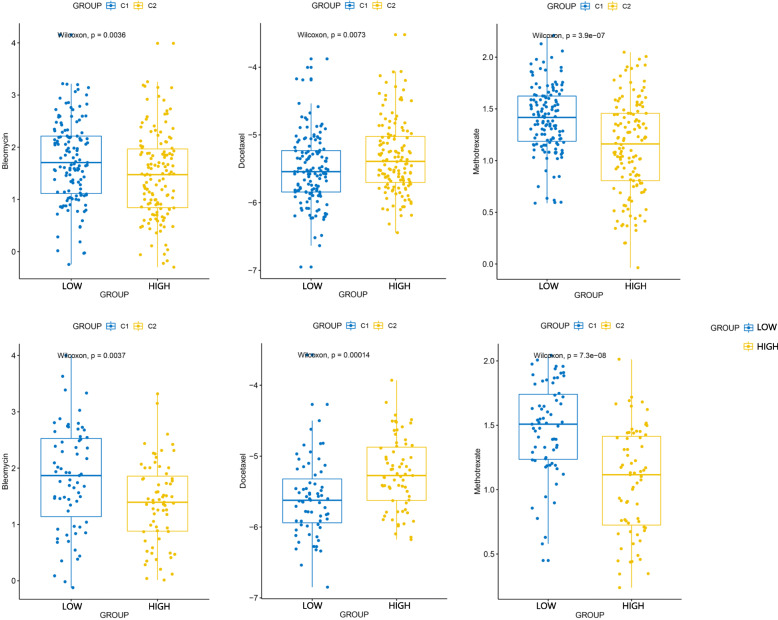
The box plots of the estimated IC50 for Bleomycin, Docetaxel and Methotrexate and showed a posetive association between hub gene expression and the response of Bleomycin and Methotrexate therapy. This relation was consistent across all two data sets. (upper:GSE65858, lower:GSE40774)

**Fig. 10:**
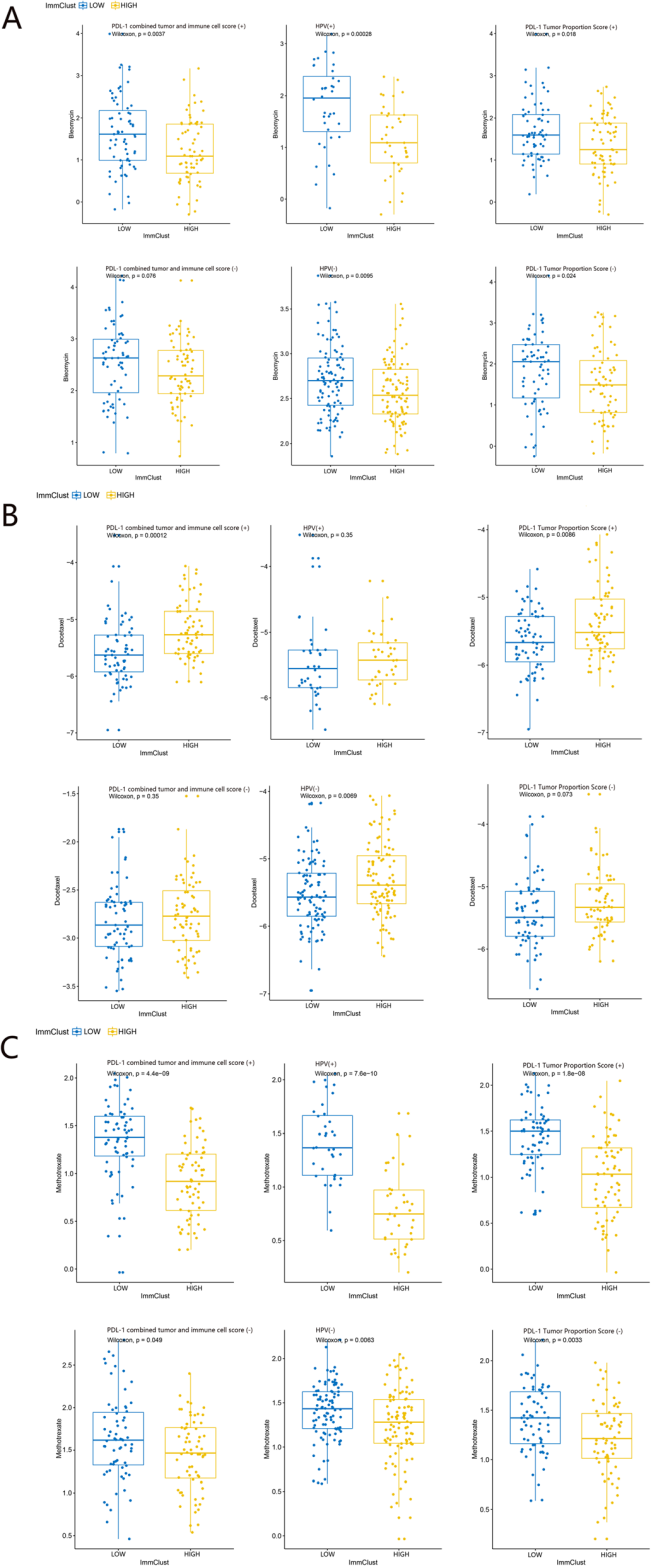
The results of chemotherapeutic response prediction indicated that the hub genes could predict the response of **a**) bleomycin and **c**) methotrexate independently. However, the response of **b**) docetaxel might be influenced by the subgroups. (upper:GSE65858, lower:GSE40774)

The assessment of immune cell function lead to the result that hub genes expression may

indicate the condition of HPV infection associated immune microenvironment. To explore its relation in clinical therapeutic strategies, we used TIDE21 and subclass mapping [22] algorithm to analyze the ICB treatment response of population with high hub genes expression. In Fig. 7b, c, d, the relation between hub genes expression and PD1 targeting therapy response showed a significant trend with increasing expression of hub genes. (*P* < 0.05) This relation was consistent across all three independent data sets. We also applied a multivariate analysis of hub genes in order to investigate whether they could add more imformation to the already existing criteria for therapy decision making. The result of multivariate analysis showed that these hub gene marker have a stable performance in ICB prediction regardless of criteria subgroups (Fig. 8). The result of chemotherapeutic response prediction is shown in Fig. 9. Among all the drugs, we found a positive and robust association between hub gene expression and the response of bleomycin and methotrexate. Subgroup analysis was performed, resulting in that the hub genes could predict the response of bleomycin and methotrexate independently in all subgroups. However, the response of docetaxel might be influenced by these subgroups (Fig. 10).

## Discussions

In this study, we used a combination of several bioinformatics tools, including WCGNA, ssGSEA, CIBERSORT, TIDE,etc., to explore significant genes both related to HPV infection status and immune cell infiltration in HNSCC patients. A total of eight hub genes was identified, including LTB, CD19, CD3D, SKAP1, KLRB1, CCL19, TBC1D10C and ARHGAP4. By analyzing downloaded single cell sequencing data, we identified the underlying function of these hub genes. We found that in the HNSCC population, the hub genes had a stable co-expression, which was related to immune cell infiltration, especially CD8+ T cells, and the infiltrative cells were in a dysfunctional status, which had corresponded with several previous studies [23, 24]. HPV + ve. Samples with high hub genes expression presented with better response to immune check point block (ICB) therapy, bleomycin and methotrexate.

In total we got 8 hub genes, which were LTB, CD19, CD3D, SKAP1, KLRB1, CCL19, TBC1D10C, ARHGAP4. Previous studies has researched on some of these genes respectively. LTB gene encodes a type II membrane protein of the TNF family lymphotoxin-β. Hsu DS [25] has reported that LTB Interacts with Methylated EGFR to mediate acquired resistance to Cetuximab in HNSCC. KLRB1 protein is expressed by NK cells and may be involved in the regulation of NK cell function. Previous studies shows that high expression of KLRB1 is closely related to the better prognosis of HNSCC patients [26]. CCL19, as the ligand of CCR7, induces several CCR7 activated pathways in metastatic HNSCC [27–38]. TBC1D10C protein is an inhibitor of both the Ras signaling pathway and calcineurin, and is reported to be related to the immune response, inflammatory response and formation of the tumor microenvironment [39]. In our research, we developed a combination of these eight genes, in which the hub genes had a stable co-expression in HNSCC population among different sample sets. The co-expression of hub genes may indicate immune microenvironment of HNSCC patients. Besides, some studies also found other underlying genetic markers. Previous studies [40, 41] demonstrated the relation of NF-kappaB proteins and AP-1 super-family proteins with oral cancer. An activation and differential expression of NF-kappaB proteins in HPV infected oral cancer was found, especially p50 and p65, leading to heterodimerization of p50/p65, which may be related to improved differentiation and better prognosis. P50:p50/NF-kappaB homodimer formation may has a cross-talk together with overexpression of AP-1 pathways in oral cancers. The relation of NF-κB proteins and AP-1 family proteins with HPV infection in tongue squamous cell carcinoma (TSCC) was mentioned by Gupta S et al. [42, 43] as well. Further exploration of the association between hub genes and NF-κB proteins, AP-1 family proteins need to be applied.

We found that expression of hub genes has a positive association with T cell dysfunction in tumor. HPV + ve HNSCC population has been found to have a typical T cell infiltration in, especially CD4+ and CD8+ T cells [14–16], and feature a high level of immune cells infiltration but high degrees of immunosuppression [23, 24, 44]. Our result corresponded previous studies. The hub genes we found related to CD8+ T cell infiltration in HPV + ve HNSCC, and the infiltrative immune cells were in dysfunction status.

While HPV infection status could divide HNSCC patients into two clinically, genetically, and immunologically different subtypes, current treatment for both subtypes are still similar [10]. Immune cell infiltration have been shown to be significant in the progression of HPV + ve HNSCC and in the favorable prognosis of HPV + ve HNSCC. Thus, ICB therapy could be a more promising treatment. HPV + ve HNSCC was reported to express higher PD-L1 proteins compared with HPV-ve HNSCC [45]. Based on this situation, we further assessed PD1/PD-L1 targeting therapy response in HPV + ve HNSCC patients. The TIDE result showed that hub genes expression was significantly associated with the activation of CD274(PD-L1), and the subclass mapping result was similar. This suggested that HPV + ve HNSCC population may be more suitable to ICB treatment. We also predicted the chemotherapy response of patients with high hub gene expression. Among all common used drugs, we found a positive association between HPV infection and response to bleomycin and methotrexate, which may be related to the better prognosis of HPV + VE HNSCC. To summarize, the eight hub genes we found stably co-express in immune cell infiltration of HPV + ve HNSCC population. The co-expression of hub genes related to an immune microenvironment featuring an increase in immune cells, especially CD8+ T cells, but high degree of immune dysfunction status. Patients with high hub gene expression had a better response to ICB treatment, bleomycin and methotrexate. The concrete mechanism of hub genes function demands further exploration.

This study has several strengths. We applied a combination of several bioinformatic tools to explore the underlying genetic mechanism of the better prognosis in HPV + ve HNSCC. Besides, we found the relation of hub genes with therapeutic response, which may help with immunotherapy and chemotherapy chosen. The weaknesses are as follow: Firstly, different prognosis may exist among HPV + ve samples with different hub genes expression. However, due to the incidence of HPV negative HNSCC is higher, we didn’t find an appropriate cohort containing enough HPV positive samples with survival information to draw a reliable conclusion. Secondly, hub genes expression has positive association with T cell dysfunction leading to treatment failure and tumor progression, but in contrast HPV + ve HNSCCs show better prognosis following standard treatment. The correlation between T cell dysfunction and the better prognosis of HPV + ve HNSCC toward standard treatment should be further studied. Finally, the relation between hub genes expression and response of docetaxel was still unclear according to our study. Since docetaxel has been part of one-line therapy for HNSCC, further research is needed to explore the possibility of hub genes expression in predicting the response to docetaxel.

## Conclusions

The eight hub genes we found presented with a stable co-expression in immune cell infiltration of HPV + ve HNSCC population. The co-expression of hub genes related to an immune microenvironment featuring an increase in immune cells but high degree of immune dysfunction status. Patients with high hub gene expression had a better response to ICB treatment, bleomycin and methotrexate. The co-expression of hub genes may be related to immune infiltration status in patients. The concrete molecular mechanism of hub genes function demands further exploration.

## Supplementary information

**Additional file 1.**

## Data Availability

The datasets used and/or analysed during the current study are available from the corresponding author on reasonable request.

## Abbreviations

HNSCC: Head and neck squamous cell carcinoma
HPV: Human papillomavirus
TME: Tumor microenvironment
TICs: Tumor-infiltrating immune cells
DCs: Dendritic cells
OS: Overall survival
WGCNA: Weighted gene co-expression network analysis
ESTIMATE: Estimation of STromal and Immune cells in MAlignant Tumor tissues Expression using data
ssGSEA: Single sample Gene Set Enrichment analysis
TIDE: Tumor Immune Dysfunction and Exclusion
GEO: Gene Expression Omnibus
DEGs: Differently expressed genes
TCGA: The Cancer Genome Atlas Project database
NK cells: Natural killer cells
TOM: Topological overlap measure
MEs: Module eigengenes
GS: Gene significance
MS: Module significance
GSEA: Gene Set Enrichment Analysis
EMT: Epithelial to mesenchymal
MM: Module membership
GDSC: Genomics of Drug Sensitivity in Cancer
IC50: Half-maximal inhibitory concentration
ICB: Immune checkpoint blockade
GSVA: Gene Set Variation Analysis

## References

[1] Argiris A, Karamouzis MV, Raben D, Ferris RL (2008). Head and neck cancer. Lancet.

[2] Adelstein D (2017). NCCN guidelines insights: head and neck cancers, version 2.2017. J Natl Compr Cancer Netw.

[3] Parkin DM, Bray F, Ferlay J, Pisani P (2005). Global cancer statistics, 2002. CA Cancer J Clin.

[4] Bozec A, Peyrade F, Fischel JL, Milano G (2009). Emerging molecular targeted therapies in the treatment of head and neck cancer. Expert Opin Emerg Drugs.

[5] Andersen AS, Koldjaer Solling AS, Ovesen T, Rusan M (2014). The interplay between HPV and host immunity in head and neck squamous cell carcinoma. Int J Cancer.

[6] Gillison ML (2000). Evidence for a causal association between human papillomavirus and a subset of head and neck cancers. J Natl Cancer Inst.

[7] Chaturvedi AK, Engels EA, Anderson WF, Gillison ML (2008). Incidence trends for human papillomavirus-related and -unrelated oral squamous cell carcinomas in the United States. J Clin Oncol.

[8] Nasman A (2015). Incidence of human papillomavirus positive tonsillar and base of tongue carcinoma: a stabilisation of an epidemic of viral induced carcinoma?. Eur J Cancer.

[9] Hammarstedt L (2006). Human papillomavirus as a risk factor for the increase in incidence of tonsillar cancer. Int J Cancer.

[10] Maxwell JH, Grandis JR, Ferris RL (2016). HPV-associated head and neck cancer: unique features of epidemiology and clinical management. Annu Rev Med.

[11] Verhaak RG (2010). Integrated genomic analysis identifies clinically relevant subtypes of glioblastoma characterized by abnormalities in PDGFRA, IDH1, EGFR, and NF1. Cancer Cell.

[12] Fakhry C (2008). Improved survival of patients with human papillomavirus-positive head and neck squamous cell carcinoma in a prospective clinical trial. J Natl Cancer Inst.

[13] Ragin CC, Taioli E (2007). Survival of squamous cell carcinoma of the head and neck in relation to human papillomavirus infection: review and meta-analysis. Int J Cancer.

[14] Lechien JR (2019). Impact of HPV infection on the immune system in oropharyngeal and non-oropharyngeal squamous cell carcinoma: a systematic review. Cells.

[15] Wang HF (2019). The double-edged sword-how human papillomaviruses interact with immunity in head and neck cancer. Front Immunol.

[16] Canning M (2019). Heterogeneity of the head and neck squamous cell carcinoma immune landscape and its impact on immunotherapy. Front Cell Dev Biol.

[17] Solomon B, Young RJ, Rischin D (2018). Head and neck squamous cell carcinoma: genomics and emerging biomarkers for immunomodulatory cancer treatments. Semin Cancer Biol.

[18] Yoshihara K (2013). Inferring tumour purity and stromal and immune cell admixture from expression data. Nat Commun.

[19] Newman AM (2015). Robust enumeration of cell subsets from tissue expression profiles. Nat Methods.

[20] Barbie DA (2009). Systematic RNA interference reveals that oncogenic KRAS-driven cancers require TBK1. Nature.

[21] Jiang P (2018). Signatures of T cell dysfunction and exclusion predict cancer immunotherapy response. Nat Med.

[22] Hoshida Y, Brunet J-P, Tamayo P, Golub TR, Mesirov JP (2007). Subclass mapping: identifying common subtypes in independent disease data sets. PLoS One.

[23] Krishna S (2018). Human papilloma virus specific immunogenicity and dysfunction of CD8(+) T cells in head and neck cancer. Cancer Res.

[24] Hladikova K (2018). Dysfunction of HPV16-specific CD8+ T cells derived from oropharyngeal tumors is related to the expression of Tim-3 but not PD-1. Oral Oncol.

[25] Hsu DS (2017). Lymphotoxin-beta interacts with methylated EGFR to mediate acquired resistance to Cetuximab in head and neck cancer. Clin Cancer Res.

[26] Liu G, Zeng X, Wu B, Zhao J, Pan Y (2019). RNA-Seq analysis of peripheral blood mononuclear cells reveals unique transcriptional signatures associated with radiotherapy response of nasopharyngeal carcinoma and prognosis of head and neck cancer. Cancer Biol Ther.

[27] Guo N (2014). Chemokine receptor 7 enhances cell chemotaxis and migration of metastatic squamous cell carcinoma of head and neck through activation of matrix metalloproteinase-9. Oncol Rep.

[28] Li P (2011). Chemokine receptor 7 promotes cell migration and adhesion in metastatic squamous cell carcinoma of the head and neck by activating integrin alphavbeta3. Int J Mol Med.

[29] Li P (2010). The chemokine receptor 7 regulates cell adhesion and migration via beta1 integrin in metastatic squamous cell carcinoma of the head and neck. Oncol Rep.

[30] Liu FY (2014). CCR7 regulates cell migration and invasion through JAK2/STAT3 in metastatic squamous cell carcinoma of the head and neck. Biomed Res Int.

[31] Liu FY (2014). CCR7 regulates cell migration and invasion through MAPKs in metastatic squamous cell carcinoma of head and neck. Int J Oncol.

[32] Liu FY (2011). NF-kappaB participates in chemokine receptor 7-mediated cell survival in metastatic squamous cell carcinoma of the head and neck. Oncol Rep.

[33] Liu FY (2010). Mammalian target of rapamycin (mTOR) is involved in the survival of cells mediated by chemokine receptor 7 through PI3K/Akt in metastatic squamous cell carcinoma of the head and neck. Br J Oral Maxillofac Surg.

[34] Wolff HA (2011). Analysis of chemokine and chemokine receptor expression in squamous cell carcinoma of the head and neck (SCCHN) cell lines. Radiat Environ Biophys.

[35] Zhen-Jin Z, Peng L, Fa-Yu L, Liyan S, Chang-Fu S (2011). PKCalpha take part in CCR7/NF-kappaB autocrine signaling loop in CCR7-positive squamous cell carcinoma of head and neck. Mol Cell Biochem.

[36] Xu Z (2015). Chemokine receptor 7 promotes tumor migration and invasiveness via the RhoA/ROCK pathway in metastatic squamous cell carcinoma of the head and neck. Oncol Rep.

[37] Zhang Z (2017). Jak3 is involved in CCR7-dependent migration and invasion in metastatic squamous cell carcinoma of the head and neck. Oncol Lett.

[38] Zhao ZJ (2011). CCL19-induced chemokine receptor 7 activates the phosphoinositide-3 kinase-mediated invasive pathway through Cdc42 in metastatic squamous cell carcinoma of the head and neck. Oncol Rep.

[39] Song Y, Pan Y, Liu J (2019). The relevance between the immune response-related gene module and clinical traits in head and neck squamous cell carcinoma. Cancer Manag Res.

[40] Mishra A, Bharti AC, Varghese P, Saluja D, Das BC (2006). Differential expression and activation of NF-kappaB family proteins during oral carcinogenesis: role of high risk human papillomavirus infection. Int J Cancer.

[41] Mishra A, Bharti AC, Saluja D, Das BC (2010). Transactivation and expression patterns of Jun and Fos/AP-1 super-family proteins in human oral cancer. Int J Cancer.

[42] Gupta S (2015). Selective participation of c-Jun with Fra-2/c-Fos promotes aggressive tumor phenotypes and poor prognosis in tongue cancer. Sci Rep.

[43] Gupta S (2018). Constitutive activation and overexpression of NF-κB/c-Rel in conjunction with p50 contribute to aggressive tongue tumorigenesis. Oncotarget.

[44] Gildener-Leapman N, Ferris RL, Bauman JE (2013). Promising systemic immunotherapies in head and neck squamous cell carcinoma. Oral Oncol.

[45] Concha-Benavente F (2016). Identification of the cell-intrinsic and -extrinsic pathways downstream of EGFR and IFNgamma that induce PD-L1 expression in head and neck cancer. Cancer Res.

